# Celecoxib reduces hepatic vascular resistance in portal hypertension by amelioration of endothelial oxidative stress

**DOI:** 10.1111/jcmm.16968

**Published:** 2021-10-05

**Authors:** Yang Tai, Chong Zhao, Linhao Zhang, Shihang Tang, Xintong Jia, Huan Tong, Rui Liu, Chengwei Tang, Jinhang Gao

**Affiliations:** ^1^ Laboratory of Gastroenterology and Hepatology State Key Laboratory of Biotherapy West China Hospital Sichuan University Chengdu China; ^2^ Department of Gastroenterology West China Hospital Sichuan University Chengdu China

**Keywords:** cyclooxygenase‐2, liver fibrosis, liver sinusoidal endothelial cells, prostaglandins, reactive oxygen species

## Abstract

The balance between endothelial nitric oxide (NO) synthase (eNOS) activation and production of reactive oxygen species (ROS) is very important for NO homeostasis in liver sinusoidal endothelial cells (LSECs). Overexpression of cyclooxygenase‐2 (COX‐2), a major intravascular source of ROS production, has been observed in LSECs of cirrhotic liver. However, the links between low NO bioavailability and COX‐2 overexpression in LSECs are unknown. This study has confirmed the link between low NO bioavailability and COX‐2 overexpression by COX‐2‐dependent PGE2‐EP2‐ERK1/2‐NOX1/NOX4 signalling pathway in LSECs *in vivo* and *in vitro*. In addition, the regulation of COX‐2‐independent LKB1‐AMPK‐NRF2‐HO‐1 signalling pathway on NO homeostasis in LSECs was also elucidated. The combinative effects of celecoxib on diminishment of ROS via COX‐2‐dependent and COX‐2‐independent signalling pathways greatly decreased NO scavenging. As a result, LSECs capillarisation was reduced, and endothelial dysfunction was corrected. Furthermore, portal hypertension of cirrhotic liver was ameliorated with substantial decreasing hepatic vascular resistance and great increase of portal blood flow. With the advance understanding of the mechanisms of LSECs protection, celecoxib may serve as a potential therapeutic candidate for patients with cirrhotic portal hypertension.

## 1 INTRODUCTION

Liver cirrhosis, characterised by diffuse fibre deposition and vascular remodelling, is a common outcome of various chronic liver diseases. Portal hypertension (PHT) is a severe consequence of liver cirrhosis that leads to life‐threatening complications with significant morbidity and mortality.^1^ Increased hepatic vascular resistance (HVR) has been proved as a primary contribution (70%~80%) to the development of PHT.^2^ Besides structural disturbances due to chronic inflammation and fibrosis, the enhanced HVR is also related to the dysfunction of liver sinusoidal endothelial cells (LSECs),^3^ which contributes up to 20%~30% of the total increased resistance in cirrhosis.^4^ Although the placement of a transjugular intra‐hepatic portosystemic shunt is extremely effective in controlling the major complications of PHT,^5^ scientists and clinicians have been trying early pharmacological reversal of cirrhotic PHT.^6^

Nitric oxide (NO) is the natural ligand for soluble guanylate cyclase and is responsible for an increase in cyclic guanosine monophosphate (cGMP), the final agent responsible for the relaxation of the vascular wall through the extrusion of cytosolic Ca^2+^. Endothelial‐derived NO, synthesised from L‐arginine by endothelial NO synthase (eNOS), plays a crucial role in maintaining endothelial homeostasis and vascular tone under physiological conditions.^7^ In the cirrhotic liver, there is a reduced NO bioavailability that plays a major role in the activation of peri‐sinusoidal hepatic stellate cells (HSCs), LSECs capillarisation, increasing intra‐hepatic vascular resistance and thereby worsening PHT.^8^, ^9^ Dysfunction of LSECs not only increases intra‐hepatic resistance and portal pressure (PP) but also remodels liver sinusoid. The balance between eNOS activation and the production of reactive oxygen species (ROS) is very important for NO homeostasis in LSECs.^10^ ROS, one of oxidative stress production, can scavenge NO by superoxide ($O_{2}^{‐}$). Either overproduction of ROS or diminished antioxidant activities may cause the redox imbalance.^11^

Cyclooxygenase (COX) is a key rate‐limiting enzyme that can catalyse the conversion of arachidonic acid (AA) to prostanoids.^12^ Among its isoforms, the constitutive COX‐1 is involved primarily in housekeeping functions, while the inducible COX‐2 modulates various inflammatory responses. Besides low NO bioavailability in LSECs, COX‐2 overexpression in cirrhotic livers has been observed in our previous studies.^13^, ^14^ However, there is less study to link them. Interestingly, COX‐2 is considered as a major intravascular source of ROS production.^15^ As a vasoconstrictor, COX‐derived prostanoids may be involved in HVR of cirrhosis through the ROS‐NO signalling pathway.^16^, ^17^ COX‐2 inhibitors exhibit anti‐oxidative effects in hypertension, cardiac hypertrophy,^18^ hepatic ischaemia‐reperfusion injury,^19^ and sepsis.^20^ Furthermore, our previous studies have shown that celecoxib, a selective COX‐2 inhibitor, could prevent PHT by inhibiting intra‐hepatic angiogenesis,^13^, ^21^ suggesting its potential protective effects on endothelial function. On the basis of that fragmentary information, we hypothesised that there may be some links between low NO bioavailability and COX‐2 overexpression in LSECs.

This study was aimed to elucidate the unclear molecular pathways related to low NO bioavailability, ROS production and COX‐2 overexpression in LSECs of cirrhotic livers and determine the effects of celecoxib on the regulation of oxidative and anti‐oxidative processes of LSECs in PHT.

## 2 MATERIALS AND METHODS

### 2.1 Animals and experimental designs

Male *Sprague*‐*Dawley* rats, weighing 180–220 g, were obtained from the Experimental Animal Center of Sichuan University. They were randomly assigned to the control, thioacetamide (TAA), and TAA +celecoxib groups with 12 animals in each group. The fibrosis‐cirrhosis rat model was established by intraperitoneal injection of TAA (Sigma‐Aldrich) 200 mg/kg in normal saline every 3 days for 16 weeks (Figure S1). The rats in the control group were injected with an equal volume of normal saline. During the last 8 weeks, the rats in TAA +celecoxib group were fed celecoxib (20 mg/kg/day, Pfizer) by gastric gavage. The rats in TAA or control group were fed their vehicle (distilled water) in the same way. All of the rats were anaesthetised with pentobarbital (50 mg/kg intraperitoneally, Sigma‐Aldrich) 72 hours after the last TAA injection. After hemodynamic evaluation, they were sacrificed via anaesthesia overdose. Then, the liver samples were harvested. The animal procedures were approved by the Animal Use and Care Committee of West China Hospital, Sichuan University (No. 2017005A).

### 2.2 Histological examination and hydroxyproline measurement

Liver tissues fixed in 4% paraformaldehyde (PFA) were embedded in paraffin, sectioned (5 μm thickness), and stained with hematoxylin and eosin (H&E) and Sirius Red. Five randomised visual fields per section (at ×100 magnification) were selected and assessed by two experienced pathologists independently blinded to the experimental grouping. The degrees of fibrosis were evaluated by the Ishak scoring system^22^ and the percentage of fibrotic area. Additionally, hepatic hydroxyproline, a major component of collagen, was measured in 400‒500 mg liver samples using a commercial kit (Nanjing Jiancheng Bioengineering Institute), according to the manufacturer's instructions.

### 2.3 *In vivo* hemodynamic evaluation

Hemodynamic evaluation was performed under anaesthesia after overnight fasting, as described previously.^23^ Briefly, the femoral artery and portal vein were cannulated with 24G catheters to measure mean arterial pressure (MAP) and heart rate (HR), and PP, respectively. Simultaneously, a perivascular ultrasonic transit‐time flow probe connected to a flow metre (Transonic Systems) was placed around the portal vein as close as possible to the liver to measure portal blood flow (PBF). Blood pressures and flows were recorded on a multichannel computer‐based recorder (Chengdu Techman Software Co., Ltd). HVR was calculated as PP/PBF.

### 2.4 Scanning electron microscope

After hemodynamic measurement, the livers were perfused *in situ* with normal saline, followed by a fixation solution of 4% PFA containing 2.5% glutaraldehyde. Then the fixed tissues were cut into short columns, snap‐frozen in liquid nitrogen, and drubbed from the middle portion. After drying in a critical point apparatus, the samples were coated with platinum and observed under a scanning electron microscope (JEOL).

### 2.5 Measurement of cGMP levels

cGMP, a marker of NO bioavailability, was determined in liver homogenates using an enzyme immunoassay (NewEast Biosciences) according to the manufacturer's instructions. Total protein concentration was quantified by the BCA assay (Beyotime Institute of Biotechnology), and cGMP levels were normalised to total protein concentration.

### 2.6 Measurement of hepatic $O_{2}^{‐}$ contents

Fresh liver tissues were embedded in optimum cutting temperature compound (Sakura Finetek), frozen with liquid nitrogen, and sectioned into 10 μm thick slices. *In situ* $O_{2}^{‐}$ levels were evaluated with an oxidative fluorescent dye dihydroethidium (DHE, 10 μM, Nanjing KeyGen Biotech Co., Ltd). Five visual fields per section (at ×100 magnification) were randomly selected and quantitatively analysed using Image‐Pro Plus 6.0.

### 2.7 Oxidative stress and antioxidant determination

Malondialdehyde (MDA), an aldehydic secondary product of lipid peroxidation, hydroxyl radical, hydrogen peroxide, and activities of enzymatic and non‐enzymatic antioxidants including superoxide dismutase 1 (SOD1), catalase (CAT), peroxidase (POD), glutathione peroxidase (GSH‐Px), glutathione reductases (GR), GSH and oxidised GSH (GSSG) were measured using commercial kits (Nanjing Jiancheng Bioengineering Institute), according to the manufacturer's instructions. All oxidative and anti‐oxidative parameters were normalised to total protein concentration.

### 2.8 Targeted metabolomic analysis of AA

Major metabolites of AA through COX‐2 pathway in liver homogenates were analysed by the Shanghai Institute of Nutrition and Health, Chinese Academy of Sciences, according to the published protocols using liquid chromatography‐mass spectrometry (LC‐MS).^24^ The radioimmunoassay for serum prostaglandin E2 **(**PGE2) was performed by the Beijing Sino‐UK Institute of Biological Technology.

### 2.9 Immunohistochemistry staining

Paraffin‐embedded liver sections (3 μm thickness) were deparaffinised in xylene and rehydrated with graded ethanol dilutions. Antigen retrieval was performed at high temperature under high pressure in sodium citrate buffer (10 mM, pH = 6.0) for 15 minutes. After blocking with H_2_O_2_ and 10% goat serum, the sections were incubated with primary antibodies overnight at 4°C followed by incubation with biotin‐streptavidin‐horseradish peroxidase (HRP) detection system (ZSGB‐BIO) at room temperature. Finally, the sections were stained with a solution of 3,3′‐diaminobenzidine (DAB, ZSGB‐BIO) and counterstained with hematoxylin. All primary antibodies used were listed in Table S1.

### 2.10 Quantitative real‐time PCR

Total RNA was extracted from the frozen liver tissues with RNAiso Plus (Takara), and cDNA was synthesised from total RNA (1 μg) using the RevertAid^™^ First Strand cDNA Synthesis Kit (Thermo Fisher Scientific). Then, quantitative PCR was performed in triplicate using SYBR Green qPCR Master Mix (Bimake) on the CFX96 Real‐Time PCR Detection System (Bio‐Rad). The mRNA expression was normalised against glyceraldehyde‐3‐phosphate dehydrogenase (GAPDH) and was shown as fold changes relative to the control group. The primer sequences were listed in Table S2.

### 2.11 Western blot analysis

Whole proteins from liver tissues or cultured cells were extracted using a protein extraction kit (Nanjing KeyGen Biotech Co., Ltd). Equal amounts of proteins for each sample were resolved by 10% or 12% SDS‐PAGE, transferred to PVDF membrane (Merck Millipore), and blocked with 5% non‐fat dry milk before being incubated with primary antibodies overnight at 4°C. Then, the blots were washed and incubated with HRP‐conjugated secondary antibodies (1:20000, ZSGB‐BIO) at room temperature for 2 hours. Protein bands were visualised by chemiluminescence using Western Blotting Luminol Reagent (Santa Cruz Biotechnology), and densitometric analyses were performed using Quantity One 4.6.2 (Bio‐Rad). Protein levels were normalised against GAPDH and were shown as fold changes relative to the control group. All primary antibodies used were listed in Table S1.

### 2.12 Biochemical analysis

Serum total bilirubin (TB), alanine aminotransferase (ALT), aspartate aminotransferase (AST), albumin (ALB), urea nitrogen (UREA), and creatinine (CREA) were detected by the WestChina‐Frontier PharmaTech Co., Ltd.

### 2.13 Cell culture and treatments

Freshly isolated LSECs were unstable in our preliminary experiments due to spontaneous capillarisation and short survival. SK‐Hep1, an immortalised human LSECs line, can maintain endothelial phenotype persistently without reagent induction.^25^, ^26^ It was obtained from the Procell Life Science and Technology Co., Ltd. The cells were cultured in Dulbecco's modified Eagle's medium (DMEM, HyClone) supplemented with 10% fetal bovine serum (FBS, Biological Industries), and 100 U/ml penicillin and 100 μg/ml streptomycin (HyClone) in a humidified atmosphere at 37°C with 5% CO_2_ in the air.

SK‐Hep1 cells were serum‐starved with 1% FBS for 16 hours before treatments. All treatments were performed in serum‐free DMEM medium. Celecoxib (1, 5, 10 μM, Selleck Chemical), 2,5‐Dimethyl‐celecoxib (DMC, a structural analogue of celecoxib with no COX‐2‐inhibiting activity, 1, 5, 10 μM, Sigma‐Aldrich), PGE2 (0.2, 1, 5 μM, MedChem Express), E‐prostanoid receptor 2 (EP2) antagonist (TG4‐155, 10 μM, MedChem Express), and ERK1/2 inhibitor (AZD6244, 1 μM, Selleck Chemical) were dissolved in dimethyl sulfoxide (DMSO, Sigma‐Aldrich). The final concentration of DMSO was 0.1% in the treated cells.

### 2.14 Plasmid transfection

COX‐2 overexpression plasmid (COX‐2‐pIRES2, Hanbio Biotechnology Co., Ltd) and its control plasmid (Empty‐pIRES2, Hanbio Biotechnology Co., Ltd) were transfected into SK‐Hep1 cells with LipoFiter (Hanbio Biotechnology Co., Ltd) following the manufacturer's protocols. Briefly, SK‐Hep1 cells in the logarithmic growth phase were seeded on 6‐well plates with a density of 5 × 10^5^ per well. COX‐2‐pIRES2 (4 μg) or Empty‐pIRES2 DNA (4 μg) and Lipofilter (12 μl) were added to each well when cells were grown to approximately 50%~70% confluence. The serum‐free medium was subsequently replaced with a complete fresh medium after 6 hours of transfection and cultured for additional 48 hours. Overexpression of COX‐2 was verified by qRT‐PCR and Western blot in mRNA and protein levels, respectively.

### 2.15 RNA interference

siRNAs targeting human AMPKα (AMPKα‐siRNA, 5′‐GAAGGUUGUAAA CCCAUAUdTdT‐3′, 5′‐AUAUGGGUUUACAACCUUCdTdT‐3′) and NRF2 (NRF2‐siRNA, 5′‐GCAGUUCAAUGAAGCUCAAdTdT‐3′, 5′‐UUGAGCUUCAUU GAACUGCdTdT‐3′) were designed and synthesised by the Hanbio Biotechnology Co., Ltd. Non‐targeting siRNA (Ctrl‐siRNA, 5′‐UUCUCCGAACGUGUCACGUdTdT‐3′, 5′‐ACGUGACACGUUCGGAGAAdTdT‐3′) was used as a negative control. SK‐Hep1 cells were plated on 6‐well plates and grown to 40%~50% confluence before transfection. Then transfection solution containing 200 μl serum‐free medium, 12 μl RNAFit (Hanbio Biotechnology Co., Ltd), and 50 nM siRNA was added to each well and incubated for 48 hours followed by subsequent experiments.

### 2.16 Measurement of intracellular $O_{2}^{‐}$ contents *in vitro*

SK‐Hep1 cells were cultured on coverslips at the bottom of 24‐well plates and incubated with corresponding treatments. DHE was added at a concentration of 5 μM and incubated at 37°C for 1 hour in the dark. Then the cells were washed by phosphate buffer, fixed with 4% PFA, and mounted with the anti‐fading medium. *In situ* $O_{2}^{‐}$ levels were determined by the fluorescence intensity.

### 2.17 Statistical analysis

All data were expressed as mean ±standard deviation (SD) and analysed using SPSS 19.0 software (SPSS). For multiple‐group comparison, one‐way ANOVA followed by Students‐Newman‐Keuls (SNK) post hoc test was performed. A *p*‐value <0.05 was considered statistically significant.

## 3 RESULTS

### 3.1 Celecoxib attenuated liver cirrhosis by inhibition of HSCs activation

**FIGURE 1:**
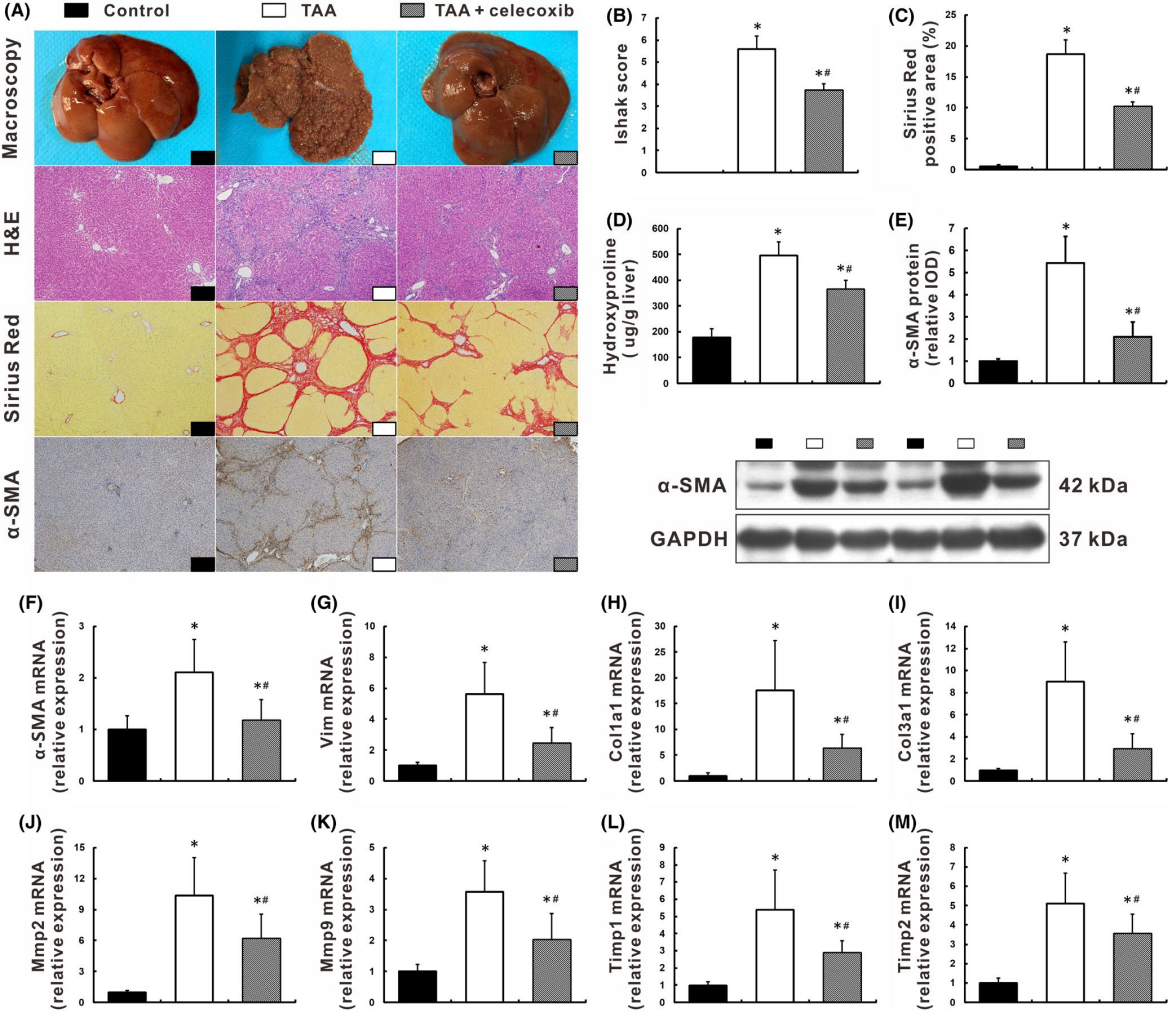
Celecoxib attenuated liver cirrhosis by inhibition of HSCs activation. Rats were repetitively exposed to TAA for 16 weeks, and celecoxib administration started after 8 weeks when the advanced fibrosis had been established. (A‐D) Representative liver morphology, histology with H&E and Sirius Red staining, immunohistochemistry of α‐SMA (A, ×100 magnification), and quantification of hepatic fibrosis as measured by the Ishak score (B), fibrotic area (C), and hydroxyproline content (D). E: Expression of α‐SMA was determined by Western blot. F‐M: Hepatic mRNA levels of HSCs activation and profibrotic genes were measured by qRT‐PCR. Data are presented as mean ±SD. n=12/group, *p < 0.05 vs. Control group, ^#^ *p* < 0.05 vs. TAA group. α‐SMA, alpha‐smooth muscle actin; Col1a1, collagen type I alpha 1; Col3a1, collagen type III alpha 1; GAPDH, glyceraldehyde‐3‐phosphate dehydrogenase; H&E: hematoxylin and eosin; HSCs, hepatic stellate cells; Mmp, matrix metalloproteinase; qRT‐PCR, quantitative real‐time PCR; TAA, thioacetamide; Timp, tissue inhibitor of metalloproteinase; Vim, vimentin

As expected, cirrhotic livers with extensive collagen deposition and pseudolobule formation were harvested from TAA group (Figure 1A). Impressively, celecoxib attenuated the cirrhotic lesions with significant lower Ishak score (3.73 ± 0.30 vs. 5.60 ± 0.57, *p* < 0.05) and fibrotic area (10.24 ± 0.74% vs.18.61 ± 2.29%, *p* < 0.05) than those in TAA group (Figure 1A‐C). Consistently, hepatic hydroxyproline, a major component of the collagen, was up‐regulated in TAA‐cirrhotic livers but was significantly decreased after celecoxib treatment, 494.70 ± 54.06 vs. 366.20 ± 33.19 μg/g wet liver, *p* < 0.05 (Figure 1D). These changes were associated with the inhibition of HSCs activation, as shown by a significant reduction in alpha‐smooth muscle actin (α‐SMA) expression in TAA +celecoxib group (Figure 1A, E). In support of this, hepatic mRNA expression of HSCs activation and profibrotic genes (*α*‐*SMA*, *Vim*, *Col1a1*, *Col3a1*, *Mmp2*, *Mmp9*, *Timp1*, *and Timp2*) were also decreased in rats treated with celecoxib, *p* < 0.05 (Figure 1F‐M).

Although body weight of each animal group was gradually increased during the experimental period. TAA‐cirrhotic rats had lower body weight gain than control rats, whereas a significant improvement was observed after celecoxib treatment (Table S3). There was no significant difference in serum TB, ALT, AST, ALB, UREA, and CREA among the three groups (Table S4), suggesting that the dose of TAA or celecoxib used in present experiment had less adverse effects on liver and renal functions.

### 3.2 Celecoxib significantly decreased PP and HVR

Cirrhotic PHT was developed in all of the rats exposed to TAA for 16 weeks (Figure S1D). PP of rats treated with celecoxib was decreased by 22.8% compared with vehicle‐treated ones, 12.51 ± 0.69 vs. 16.20 ± 0.84 cm H_2_O, *p* < 0.05 (Table 1). The attenuation of PHT was associated with a significant increase in PBF, 12.25 ± 0.75 vs. 13.83 ± 0.83 ml/min, *p* < 0.05 (Table 1), indicating a significant reduction in HVR, 1.33 ± 0.10 vs. 0.91 ± 0.05 cm H_2_O ml^−1^ min, *p* < 0.05 (Table 1). At the same time, there were no changes in HR and MAP among the three groups (Table 1). In addition, the spleen index (spleen weight/rat weight) was also decreased with celecoxib treatment, further verifying the alleviation of PHT (Table S3).

**TABLE 1:** Effects of celecoxib on hepatic and systemic hemodynamics

Parameter	Control	TAA	TAA +celecoxib
n	12	12	12
HR (beats/min)	320 ± 38	335 ± 41	331 ± 38
MAP (mm Hg)	110 ± 20	99 ± 18	101 ± 17
PP (cm H_2_O)	9.68 ± 0.57	16.20 ± 0.84^*^	12.51 ± 0.69^*,#^
PBF (ml/min)	13.18 ± 1.08	12.25 ± 0.75^*^	13.83 ± 0.83^#^
HVR (cm H_2_O/ml/min^−1^)	0.74 ± 0.04	1.33 ± 0.10^*^	0.91 ± 0.05^*,#^

Note

Data are presented as mean ±SD. *p < 0.05 vs. Control group, ^#^ *p* < 0.05 vs. TAA group.

Abbreviations: HR, heart rate; HVR, hepatic vascular resistance; MAP, mean arterial pressure; PBF, portal blood flow; PP, portal pressure; TAA, thioacetamide.

### 3.3 Celecoxib arrested LSECs capillarisation by increasing NO bioavailability

**FIGURE 2:**
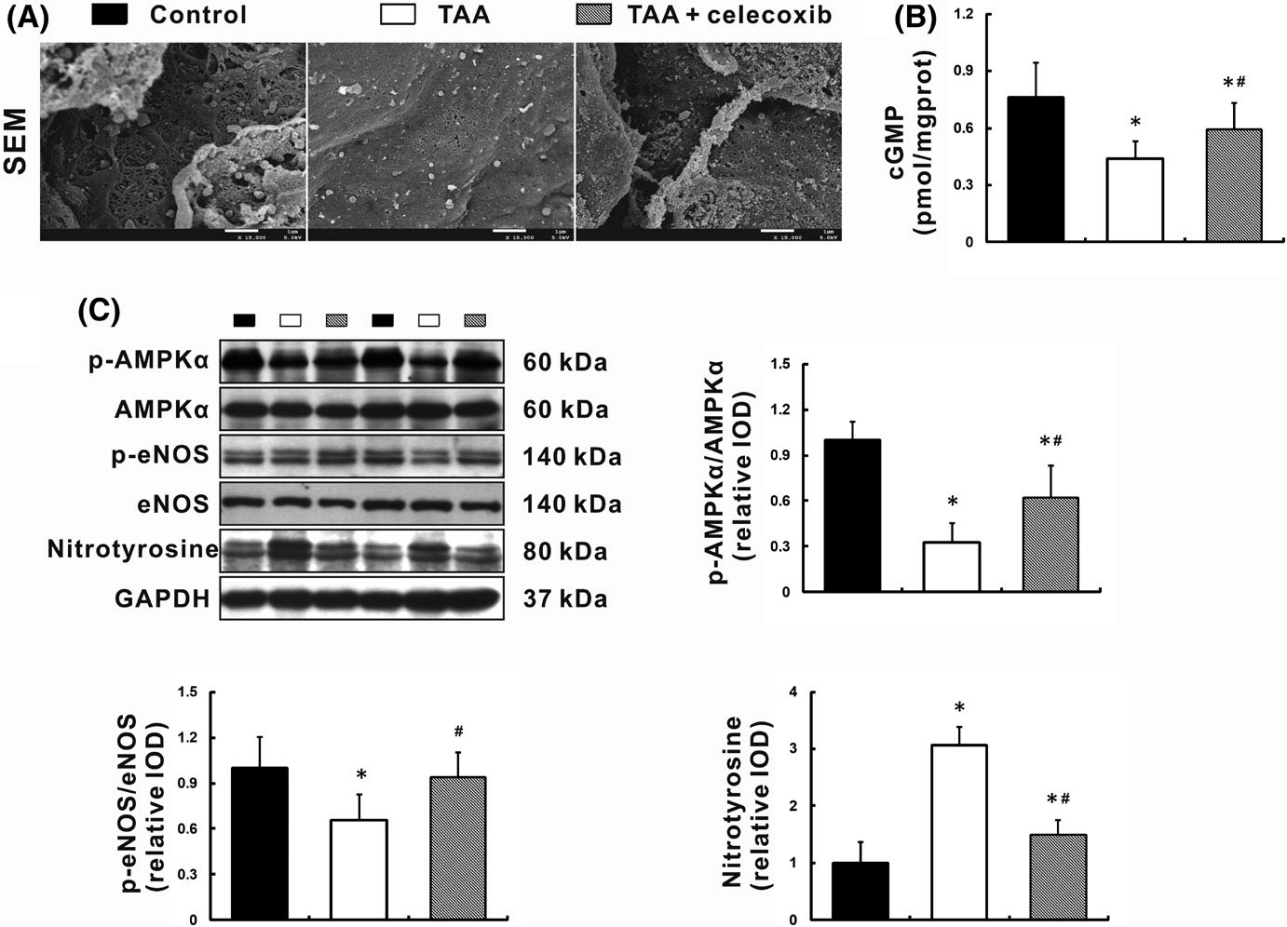
Celecoxib arrested LSECs capillarisation by increasing NO bioavailability. (A) Ultrastructure of LSECs (SEM, ×15,000 magnification). (B) NO bioavailability in liver homogenates was measured as cGMP levels. (C) Expression of key molecules in the NO metabolic pathway was determined by Western blot. Data are presented as mean ±SD. n=12/group, *p < 0.05 vs. Control group, ^#^ *p* < 0.05 vs. TAA group. AMPK, AMP‐activated protein kinase; eNOS, endothelial nitric oxide synthase; GAPDH, glyceraldehyde‐3‐phosphate dehydrogenase; LSECs, liver sinusoidal endothelial cells; NO, nitric oxide; SEM, scanning electron microscope; TAA, thioacetamide

LSECs in TAA‐cirrhotic rats underwent capillarisation with an obvious decline in the numbers of fenestrae (Figure 2A). Celecoxib attenuated the capillarisation induced by TAA (Figure 2A). Hepatic cGMP level, a marker of NO bioavailability, was significantly increased in TAA +celecoxib group compared with TAA group, *p* < 0.05 (Figure 2B). This improvement was associated with a 1.4‐fold increase in eNOS phosphorylation and a 50% decrease of nitrotyrosine expression, *p* < 0.05 (Figure 2C), indicating the counterbalance between eNOS‐induced NO synthesis and $O_{2}^{‐}$ mediated NO scavenging. Additionally, the phosphorylation of AMPKα inhibited by TAA was partially restored following celecoxib treatment (Figure 2C), which was consistent with the changes in the NO‐cGMP metabolic pathways.

### 3.4 Celecoxib reduced hepatic oxidative stress

**FIGURE 3:**
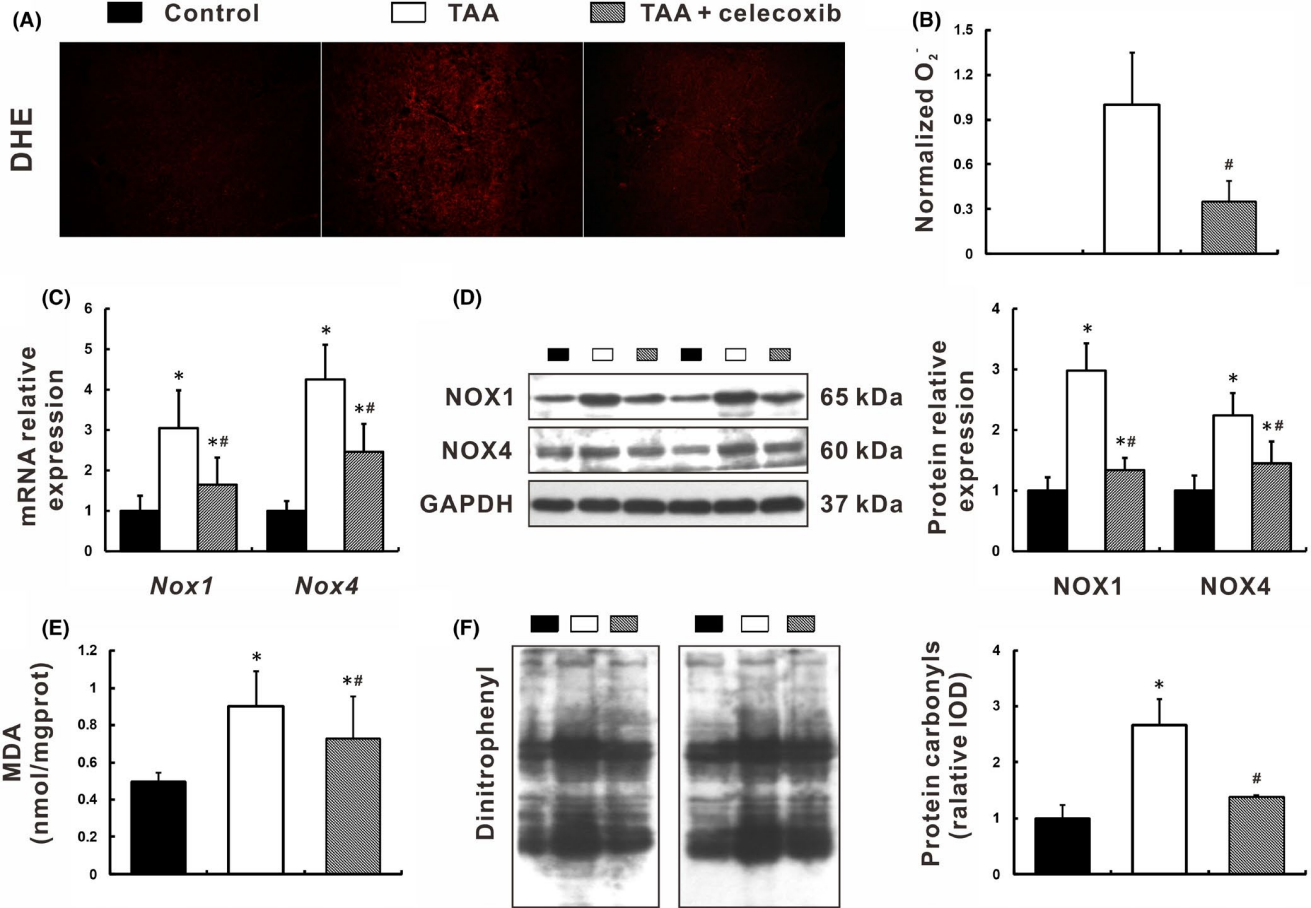
Celecoxib reduced hepatic oxidative stress. (A‐B) Hepatic oxidative stress was measured as *in situ* $O_{2}^{‐}$ levels with DHE fluorescence staining (A), and quantified by intensity analysis (B). C‐D: Expression of NOX1 and NOX4 was determined in mRNA and protein levels by qRT‐PCR (C) and Western blot (D), respectively. (E) Lipid peroxidation was measured as MDA levels. (F) Protein carbonylation was determined by dinitrophenyl expression. Data are presented as mean ±SD. n=12/group, *p < 0.05 vs. Control group, ^#^ *p* < 0.05 vs. TAA group. DHE, dihydroethidium; GAPDH, glyceraldehyde‐3‐phosphate dehydrogenase; MDA, malondialdehyde; NOX, NADPH oxidase; qRT‐PCR, quantitative real‐time PCR; TAA, thioacetamide

DHE staining, showing *in situ* superoxide anion with oxidative fluorescent dye, was very faint in control livers (Figure 3A). In contrast, numerous particles emitting red fluorescence were distributed in the hepatic sinusoid, portal area, and fibrous septum of TAA‐cirrhotic livers (Figure 3A). After treatment with celecoxib, DHE staining was significantly diminished (Figure 3A). Quantitatively, fluorescence intensity in TAA +celecoxib group was decreased by 65% compared with that in TAA group (*p* < 0.05, Figure 3B). Consistently, celecoxib administration resulted in a significant reduction in hydroxyl radical and hydrogen peroxide levels (*p* < 0.05, Figure S2A, B) and down‐regulation of NADPH oxidases (NOX1 and NOX4), the main source of liver ROS production, both in mRNA and protein levels (*p* < 0.05, Figure 3C, D). Furthermore, as indicators of lipid peroxidation and protein carbonylation, the reduction of MDA and dinitrophenyl after celecoxib treatment reflected the amelioration of oxidative damage to lipid and protein, respectively (Figure 3E, F).

### 3.5 Celecoxib restored hepatic anti‐oxidative capacity via activation of the NRF2‐HO‐1 signalling pathway

**FIGURE 4:**
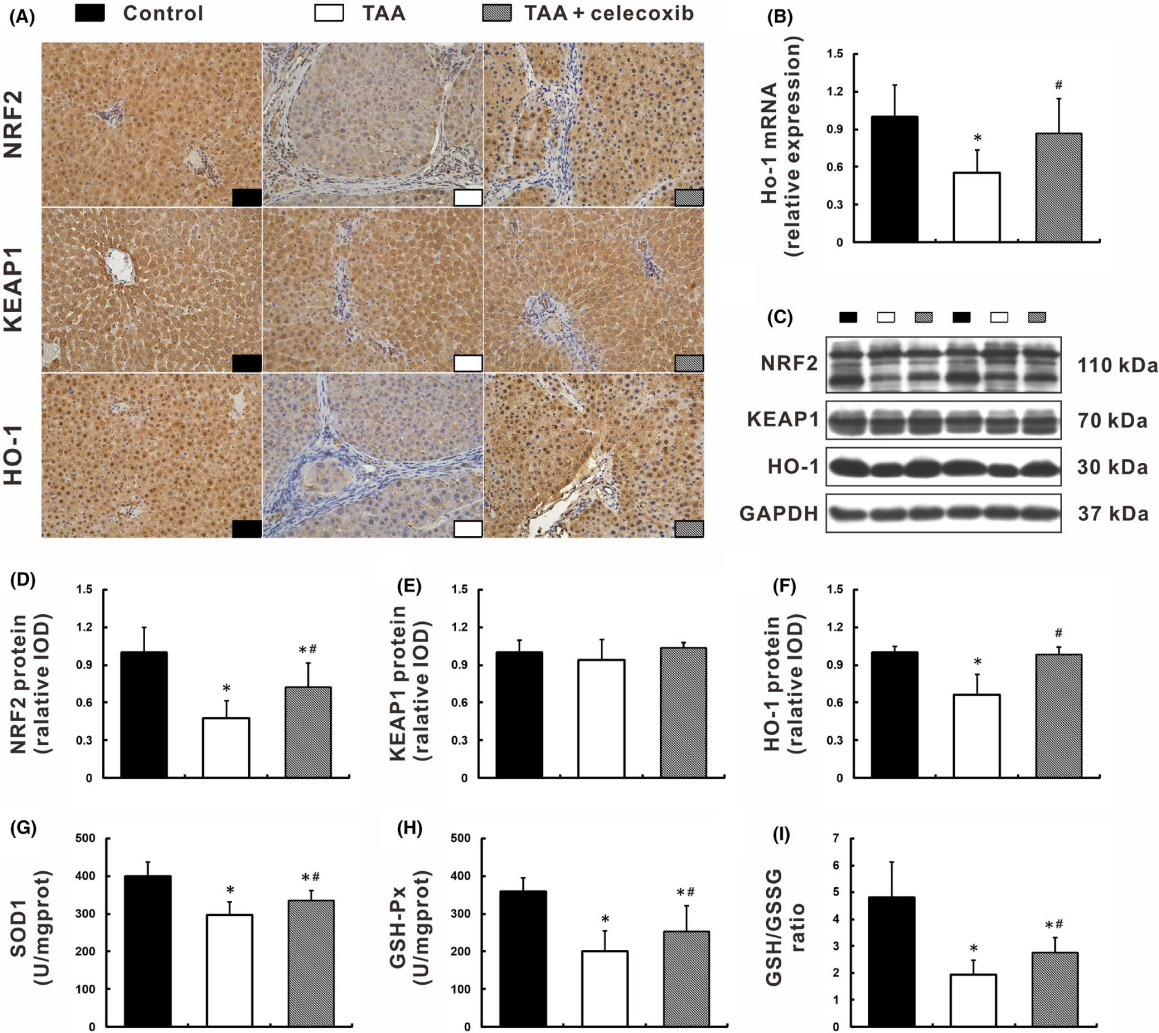
Celecoxib restored hepatic anti‐oxidative capacity via activation of the NRF2‐HO‐1 signalling pathway. (A‐C) Expression of the NRF2‐KEAP1‐HO‐1 signalling pathway was determined by immunohistochemistry (A, ×400 magnification), qRT‐PCR (B), and Western blot (C). (D‐F) Densitometry analysis of NRF2 (D), KEAP1 (E), and HO‐1 (F). G‐I: Hepatic anti‐oxidative capacity was measured as activities of enzymatic antioxidants SOD1 (G) and GSH‐Px (H), and non‐enzymatic antioxidant GSH/GSSG (I). Data are presented as mean ±SD. n=12/group, *p < 0.05 vs. Control group, ^#^ *p* < 0.05 vs. TAA group. GAPDH, glyceraldehyde‐3‐phosphate dehydrogenase; GSH‐Px, glutathione peroxidase; GSSG, oxidised glutathione; HO‐1, heme oxygenase‐1; KEAP1, Kelch‐like ECH‐associated protein 1; NRF2, nuclear factor (erythroid‐derived 2)‐like 2; qRT‐PCR, quantitative real‐time PCR; SOD1, superoxide dismutase 1; TAA, thioacetamide

Given that NRF2‐KEAP1 complex is the key regulator of anti‐oxidative signalling, we determined whether celecoxib diminished superoxide radicals generation through activation of the NRF2‐KEAP1 pathway (Figure 4A‐F). HO‐1, a downstream effector molecule of NRF2, was also determined in mRNA and protein levels. As expected, the down‐regulation of NRF2 and HO‐1 expression in TAA group was significantly inhibited by celecoxib treatment, *p* < 0.05 (Figure 4A‐D, F). The data from Westen blot showed that there was no significant difference of KEAP1 expression among the three groups (Figure 4A, C, E). Moreover, celecoxib treatment greatly enhanced the activities of a battery of anti‐oxidative enzymes and detoxification factors activated by NRF2, including SOD1, GSH‐Px, CAT, and GSH/GSSG, *p* < 0.05 (Figure 4G‐I; Figure S2C). Collectively, these findings illustrated that celecoxib restored hepatic anti‐oxidative capacity via activation of the NRF2‐HO‐1 signalling pathway.

### 3.6 Celecoxib inhibited the COX‐2‐PGE2‐EP2 signalling pathway

**FIGURE 5:**
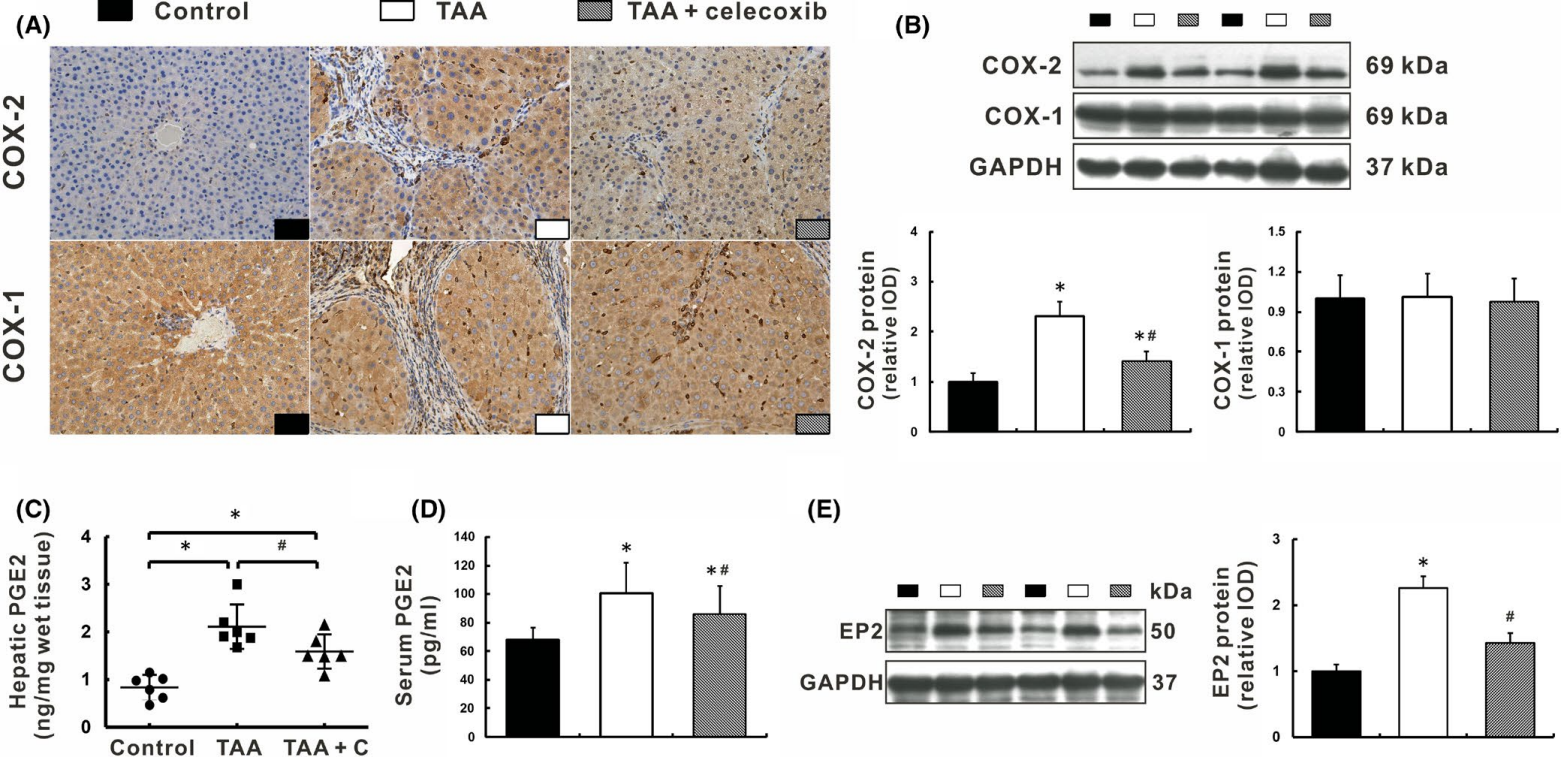
Celecoxib inhibited the COX‐2‐PGE2‐EP2 signalling pathway. (A‐B) Expression of COX‐2 and COX‐1 was determined by immunohistochemistry (A, ×400 magnification) and Western blot (B). (C‐D) PGE2 levels were measured in liver homogenates (C) and serum (D) using LC‐MS and radioimmunoassay, respectively. (E) Expression of EP2 was determined by Western blot. Data are presented as mean ±SD. n=12/group, *p < 0.05 vs. Control group, ^#^ *p* < 0.05 vs. TAA group. COX, cyclooxygenase; EP2, E‐prostanoid receptor 2; GAPDH, glyceraldehyde‐3‐phosphate dehydrogenase; LC‐MS, liquid chromatography‐mass spectrometry; PGE2, prostaglandin E2; TAA, thioacetamide

COX‐2 expression in TAA‐cirrhotic livers was significantly increased compared to control rats, *p* < 0.05 (Figure 5A, B). Celecoxib treatment significantly decreased COX‐2 expression in the liver, *p* < 0.05 (Figure 5A, B). Meanwhile, COX‐2‐derived targeted metabolomic analysis showed that PGE2 levels in both liver and serum were remarkably decreased after celecoxib treatment, *p* < 0.05 (Figure 5C, D), while other prostaglandins levels were kept normal (data not shown). Consistently, EP2 expression was down‐regulated in the livers of TAA +celecoxib group compared to that in TAA group, *p* < 0.05 (Figure 5E), indicating a direct inhibition of the COX‐2‐PGE2‐EP2 signalling pathway.

### 3.7 COX‐2 promoted ROS production through the PGE2‐EP2‐ERK1/2 signalling pathway involving up‐regulation of NOX1 and NOX4

**FIGURE 6:**
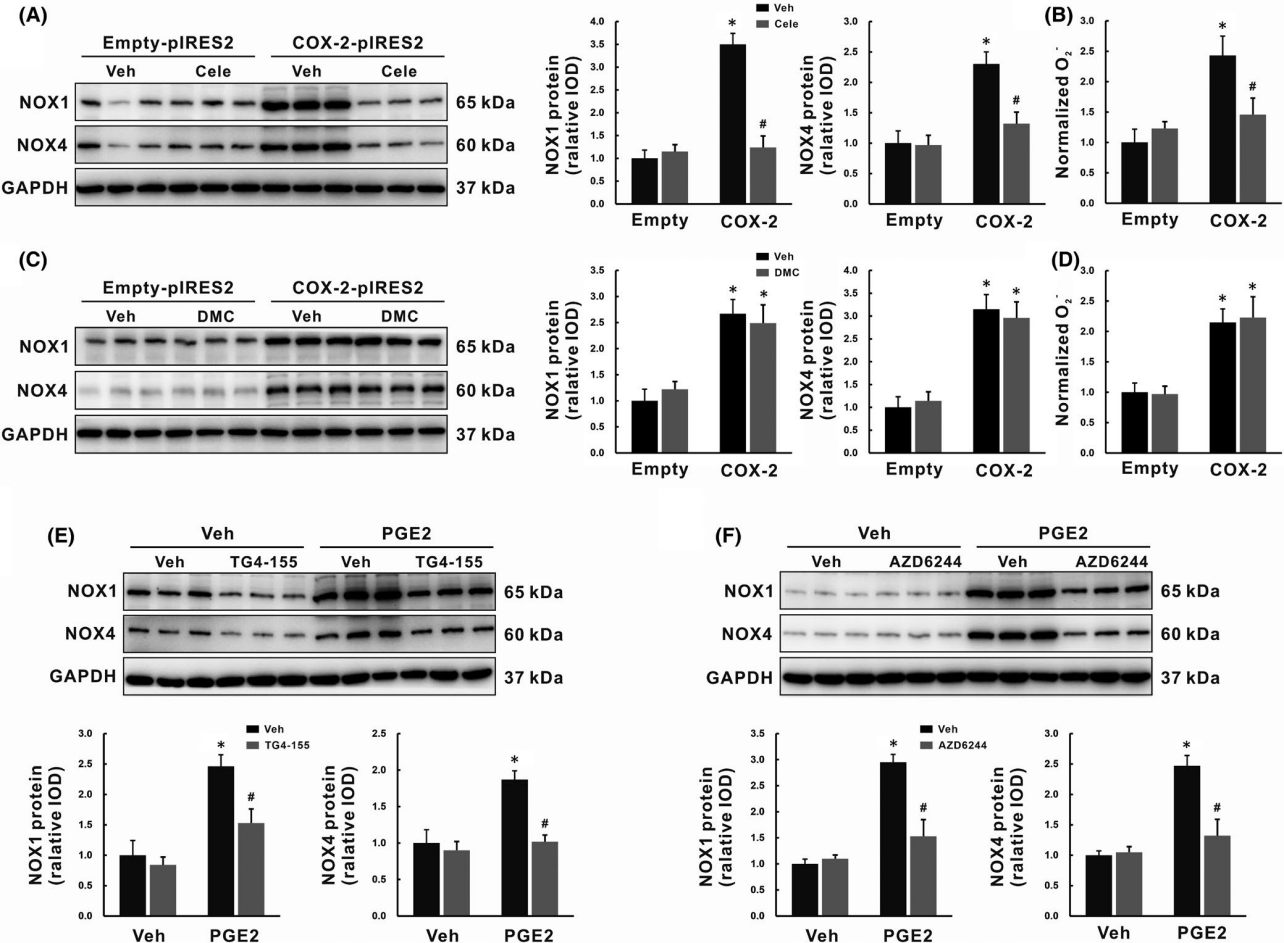
COX‐2 promoted ROS production through the PGE2‐EP2‐ERK1/2 signalling pathway involving up‐regulation of NOX1 and NOX4. (A‐B) SK‐Hep1 cells were transfected with COX‐2‐pIRES2, then cells were treated with celecoxib for 24 h. NOX1 and NOX4 protein expression were determined by Western blot (A), and basal ROS production was measured as i*n situ* $O_{2}^{‐}$ levels with DHE fluorescence staining (B). (C‐D) SK‐Hep1 cells were transfected with COX‐2‐pIRES2, then cells were treated with DMC for 24 h. NOX1 and NOX4 protein expression were determined by Western blot (C), and basal ROS production was measured as in situ $O_{2}^{‐}$ levels with DHE fluorescence staining (D). (E‐F) SK‐Hep1 cells were pretreatment with EP2 antagonist TG4‐155 (E) or ERK1/2 inhibitor AZD6244 (F) for 2 h before PGE2 treatment. NOX1 and NOX4 protein expressions were determined by Western blot. Data are presented as mean ±SD. n=3/group, *p < 0.05 vs. Vehicle or Empty‐pIRES2 group, ^#^ *p* < 0.05 vs. PGE2 or COX‐2‐pIRES2 group. Cele, celecoxib; COX‐2, cyclooxygenase‐2; DMC, 2,5‐Dimethyl‐celecoxib; EP2, E‐prostanoid receptor 2; GAPDH, glyceraldehyde‐3‐phosphate dehydrogenase; NOX, NADPH oxidase; PGE2, prostaglandin E2; ROS, reactive oxygen species; Veh, vehicle

To further validate the link between COX‐2 and NOX‐derived ROS, SK‐Hep1 cells with COX‐2 overexpression were used for *in vitro* study. This human LSECs line was transfected with a plasmid containing the open reading frame of human COX‐2 (COX‐2‐pIRES2).^25^, ^26^ Overexpression of COX‐2 in SK‐Hep1 cells was verified by qRT‐PCR and Western blot (data not shown). Compared to Empty‐pIRES2‐transfected controls, higher NOX1 and NOX4 protein levels were detected in COX‐2‐pIRES2‐transfected cells (*p* < 0.05, Figure 6A), with higher basal ROS contents (*p* < 0.05, Figure 6B). These up‐regulated proteins due to COX‐2 overexpression were suppressed by celecoxib (*p* < 0.05, Figure 6A, B), but not its inactive analogue DMC (Figure 6C, D), indicating that COX‐2 overexpression induced ROS production via up‐regulation of NOX1 and NOX4 in SK‐Hep1 cells.

PGE2 is a major product of COX‐2 enzyme activity and exerts its pro‐inflammatory action through binding to EP2 receptor. Our experiment showed that 5 μM PGE2 induced NOX1/NOX4 expression and ROS production 24 hours post‐treated (Figure S3). The expression of NOX1/NOX4 induced by PGE2 could be inhibited by pretreatment with an EP2 antagonist TG4‐155 for 2 hours, *p* < 0.05 (Figure 6E), as well as an ERK1/2 inhibitor AZD6244, *p* < 0.05 (Figure 6F). These findings suggested that COX‐2 promoted NOX‐derived ROS production through the PGE2‐EP2‐ERK1/2 signalling pathway.

### 3.8 Celecoxib induced endothelial HO‐1 via COX‐2‐independent activation of the LKB1‐AMPK‐NRF2 signalling pathway

**FIGURE 7:**
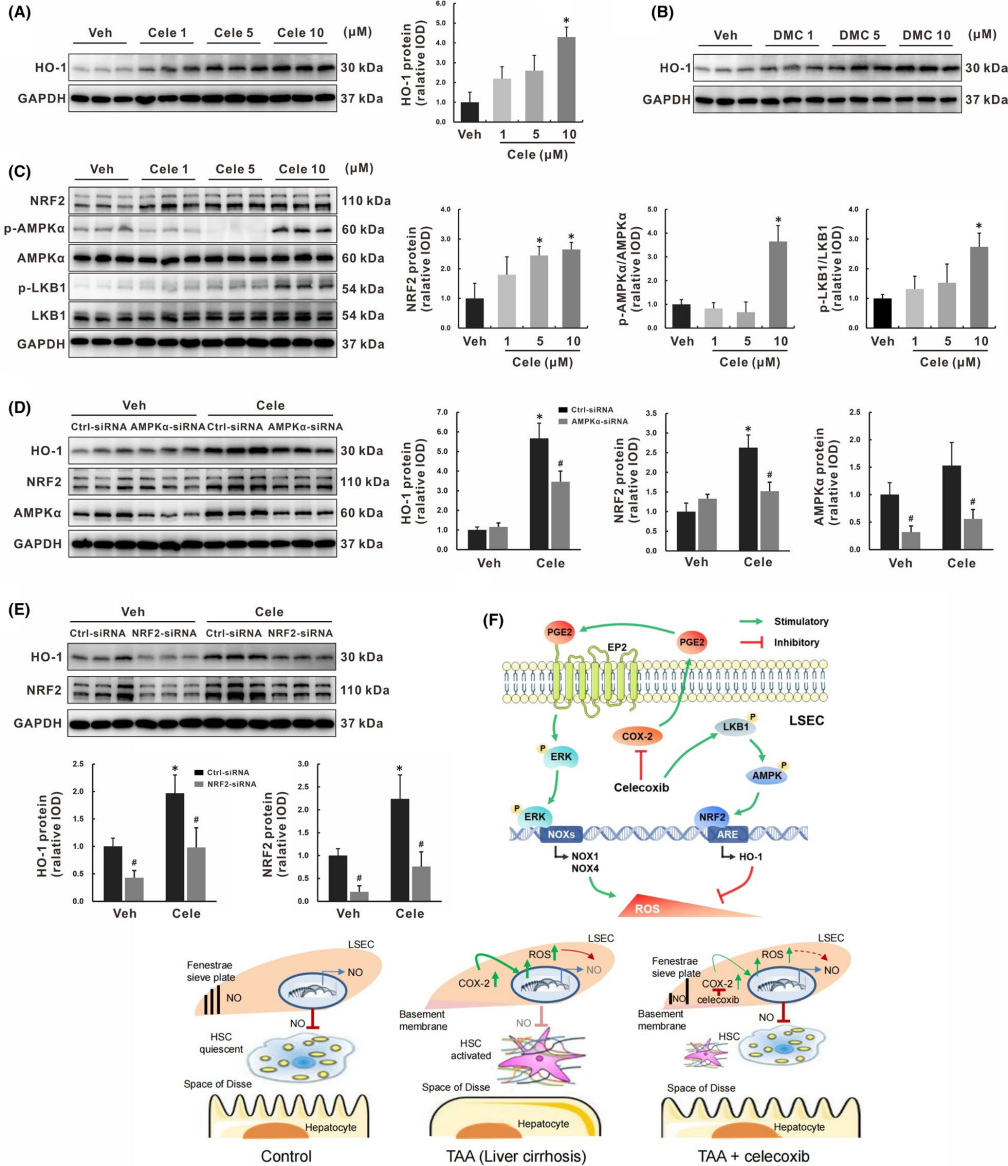
Celecoxib induced endothelial HO‐1 via COX‐2‐independent activation of the LKB1‐AMPK‐NRF2 signalling pathway. (A‐B) SK‐Hep1 cells were treated with celecoxib (A) or DMC (B) for 24 h, HO‐1 protein expression was determined by Western blot. (C) SK‐Hep1 cells were treated with celecoxib for 24 h, key molecules in the LKB1‐AMPK‐NRF2 signalling pathway were determined by Western blot. (D‐E) SK‐Hep1 cells were transfected with AMPKα‐siRNA (D) or NRF2‐siRNA (E) for 48 h, followed by induction with celecoxib (10 μM) for additional 24 h, key molecules in the AMPK‐NRF2‐HO‐1 signalling pathway were determined by Western blot. (F) The mechanism schematic model of celecoxib on LSECs protection by ameliorating endothelial oxidative stress via COX‐2‐dependent and COX‐2‐independent signalling pathways. Data are presented as mean ±SD. n = 3/group, *p < 0.05 vs. Vehicle or Control‐siRNA vehicle‐treated group, ^#^ *p* < 0.05 vs. Control‐siRNA group. AMPK, AMP‐activated protein kinase; Cele, celecoxib; Ctrl, control; DMC, 2,5‐Dimethyl‐celecoxib; GAPDH, glyceraldehyde‐3‐phosphate dehydrogenase; HO‐1, heme oxygenase‐1; LKB1: liver kinase B1; LSECs: liver sinosoidal endothelial cells; NRF2, nuclear factor (erythroid‐derived 2)‐like 2; Veh, vehicle

Both AMPK and NRF2 pathways are involved in the defence against oxidative stress. Because AMPKα and NRF2‐HO‐1 could be restored by celecoxib treatment *in vivo*, the effect of celecoxib on this pathway was also determined with SK‐Hep1 cells *in vitro*. HO‐1 protein levels were elevated by celecoxib in a concentration‐dependent manner, *p* < 0.05 (Figure 7A). When celecoxib was instead by DMC, the level of HO‐1 protein was increased 2–4 folds in a similar manner, *p* < 0.05 (Figure 7B). The response of SK‐Hep1 cells to DMC mirrored that seen with celecoxib, suggesting its induction of endothelial HO‐1 via a COX‐2‐independent mechanism.

With regarding to AMPK signalling pathway involved in the regulation of HO‐1 induction, it was supported by the following results. First, AMPKα phosphorylation and NRF2 expression in SK‐Hep1 cells were increased with celecoxib treatment in a concentration‐dependent manner, *p* < 0.05 (Figure 7C). Next, AMPKα activation was enhanced 3–5 folds by celecoxib treatment for 60 minutes or 120 minutes, whereas NFR2 and HO‐1 protein levels were not altered (Figure S4). Moreover, LKB1, the upstream activator of AMPK, was also activated by celecoxib in a concentration‐ and time‐dependent manner, as evidenced by phosphorylation at Ser^428^, *p* < 0.05 (Figure 7C and Figure S4). These findings suggested that LKB1‐AMPK pathway may modulate NRF2‐HO‐1 expression. To verify this hypothesis, SK‐Hep1 cells were transfected with AMPKα‐siRNA or NRF2‐siRNA for 48 hours, followed by induction with celecoxib (10 μM) for an additional 24 hours. Up‐regulation of HO‐1 or NRF2 expression was suppressed by silencing AMPKα before celecoxib treatment, *p* < 0.05 (Figure 7D). Similarly, the increased HO‐1 expression was significantly inhibited by siRNA‐mediated knockdown of NRF2, *p* < 0.05 (Figure 7E). Consistent with this, we also observed similar results in DMC‐induced HO‐1 expression (Figure S5), confirming the COX‐2 independency of celecoxib on the LKB1‐AMPK‐NRF2‐HO‐1 signalling pathway.

## 4 DISCUSSION

Over the past 30 years, pharmacological therapy for PHT is consisting mainly of the continued oral administration of non‐selective beta‐blockers (propranolol, nadolol, carvedilol) for the prevention of first or recurrent variceal bleeding, and on the short‐term intravenous infusion of terlipressin, somatostatin, or somatostatin analogues for acute variceal bleeding.^6^ These treatments are aimed at correcting the increased splanchnic blood flow. It is only recently that this strategy has been changed. Progress of the modulation of intra‐hepatic endothelial dysfunction has opened entirely new perspectives for developing more effective treatment.^2^

NO‐cGMP is crucial regulators for LSECs to maintain the fenestration, HSCs quiescence and portal pressure.^27^ Both insufficient syntheses of NO and increased levels of superoxide and peroxynitrite have been well documented.^8^ The present study firstly established the elaborate links between low NO bioavailability and COX‐2 overexpression in LSECs. The combinative effects of celecoxib on diminishment of ROS decreased NO scavenging. As a result, LSECs capillarisation was reduced, losing fenestrae was protected, and endothelial dysfunction was corrected. Furthermore, PHT of cirrhotic liver was ameliorated with substantial decreasing HVR and great increase of portal blood flow. Figure 7F summarised the relationship between loop of ROS regulated by celecoxib via COX‐2‐dependent or COX‐2‐independent signalling pathways and NO bioavailability in LSECs. Such improvement of LSECs remodelling and dysfunction by celecoxib was beneficial for amelioration of liver fibrosis by inhibiting HSCs activation and down‐regulating profibrotic genes.

In accordance with NO pathophysiological abnormalities, a drug that would be able to increase NO bioavailability in the liver would be hopeful to arrest liver fibrosis and decrease PP. Several efforts to improve NO bioavailability within the liver have been attempted including exogenous administration of NO donors,^28^, ^29^ transfecting the liver with adenovirus encoding eNOS,^30^ enhancing eNOS activity by simvastatin,^31^ and decreasing NO scavenging by means of antioxidants, such as metformin.^32^ Actually, liver‐targeted NO donor drugs that mimic the cellular effects of endogenous NO and specifically release in the liver have been evaluated in the treatment of PHT,^28^, ^29^ whereas they failed to reduce PP in clinical trials.^33^ Simvastatin administration significantly decreased hepatic venous pressure gradient but did not reduce HVR.^34^ Therefore, addition of simvastatin to carvedilol for 3 months for primary prophylaxis of variceal bleeding did not improve hemodynamic response over carvedilol monotherapy.^35^ Metformin caused a significant reduction in liver fibrosis and PP but no enhancement of portal blood flow.^32^ The ideal drug for PHT is pictured as one that should reduce PP by decreasing intra‐hepatic vascular resistance, while maintaining or enhancing hepatic blood flow.^36^ By this picture, celecoxib should be quite satisfactory compared with other drugs. It significantly reduced HVR and increased portal blood flow in PTH by increasing eNOS‐derived NO synthesis and reducing ROS‐mediated NO scavenging in this study. The dual function of celecoxib was obviously superior to simply NO supplement or scavenging.

The possibility that antioxidants may act as therapeutic agents in fibrosis has been extensively analysed in animal models. LSECs uptake of drugs with the ability to regulate NO bioavailability should be essential. Our study targeted COX‐2 expression in LSECs *in vivo* and in *vitro*. Unlike other biologicals like chemokines and cytokines which have a short plasma half‐life, overexpression of COX‐2 in LSECs provides a stable target for celecoxib action. Although anti‐oxidative effects of meloxicam, another COX‐2 inhibitor, have been observed in carbon tetrachloride (CCl_4_)‐induced fibrotic livers, its function remains unclear.^37^ The exact mechanisms underlying the ROS homeostasis regulation by celecoxib were elucidated through a series of *in vivo* and *in vitro* experiments. In addition, NADPH oxidase family members, such as NOX1 and NOX4, are major endogenous ROS sources and essential players in HSCs activation and LSECs apoptosis.^38^ Celecoxib functioned as a specific inhibitor of COX‐2, the key enzyme that catalyses the generation of PGs from AA, and exerted its anti‐oxidative effects by reducing NOX‐derived ROS through inhibition of the COX‐2‐PGE2‐EP2‐ERK1/2 signalling pathway. Furthermore, the NRF2‐HO‐1 anti‐oxidative pathway also plays a central role in scavenging ROS.^39^ Either celecoxib or DMC, a structural analogue of celecoxib, directly induced endothelial HO‐1 via COX‐2‐independent activation of the LKB1‐AMPK‐NRF2 signalling pathway of LSEC line *in vitro*, indicating that celecoxib still acted on the key effector cells of fibrogenesis.

Accumulated data have indicated the beneficial effects of celecoxib on various kinds of experimental fibrosis, there are several studies showing inconsistent results.^40^, ^41^, ^42^ They found that celecoxib did not prevent liver fibrosis induced by CCl_4_ or bile duct ligation. The milder lesions, inadequate doses of CCl_4_ and celecoxib at dose of 15 mg/kg as well as shorter experiment period may explain the negative results. Moreover, it is not rational for the design of a study that continuous administration of celecoxib through a subcutaneous osmotic pump kept hepatic PGE2 levels below the physiological conditions.^42^ As PGE2 has been proposed to mediate both profibrotic and antifibrotic effects of COX‐2 inhibition.^43^, ^44^ In contrast, once‐daily oral gavage of celecoxib in our study merely suppressed the elevated COX‐2‐derived PGE2 synthesis in cirrhotic livers.

COX‐2 selective inhibitors (coxibs) have been widely used in the treatment of chronic inflammatory diseases. The long‐term safety of celecoxib is still of concern. This experiment indicated that administration of celecoxib at dose of 20 mg/kg/d for 8 weeks did not show adverse effects on liver and renal functions. With regarding to the risk of cardiovascular events, increasing data indicate the clinical safety of celecoxib. The PRECISION study including 24,081 patients revealed that celecoxib at moderate doses was non‐inferior to ibuprofen or naproxen in cardiovascular safety.^45^ Furthermore, celecoxib therapy has generated significant clinical benefits for endothelial function improvement in patients with coronary artery disease and hypertension.^46^, ^47^ In addition, preliminary clinical evidence has shown that celecoxib does not increase the risk of variceal and non‐variceal upper gastrointestinal events,^48^ nor impair renal function in decompensated cirrhosis.^49^ Although celecoxib has favourable ameliorating effects against liver fibrosis and PHT, further clinical trials should be performed to evaluate its efficacy and safety.

In conclusion, celecoxib ameliorated PHT of cirrhotic liver by substantial decreasing HVR on the basis of a great increase of portal blood flow. Protection of LSECs from capillarisation and endothelial dysfunction by celecoxib was contributed to the reduction of HVR. The function of LSECs was improved by enhancing their NO bioavailability, which was the counterbalance of pro‐oxidative and anti‐oxidative machinery through both COX‐2‐dependent and COX‐2‐independent pathways. With the advance understanding of the mechanisms of LSECs protection, celecoxib may be a potential therapeutic candidate for patients with cirrhotic PHT.

## CONFLICT OF INTEREST

All authors declare no conflict of interest.

## AUTHOR CONTRIBUTIONS

**Yang Tai:** Conceptualization (equal); Data curation (equal); Formal analysis (equal); Funding acquisition (equal); Investigation (lead); Methodology (equal); Project administration (equal); Software (equal); Validation (lead); Writing‐original draft (lead). **Chong Zhao:** Data curation (equal); Investigation (equal); Methodology (equal); Resources (equal). **Linhao Zhang:** Investigation (equal); Methodology (equal). **Shihang Tang:** Methodology (equal). **Xintong Jia:** Methodology (equal). **Huan Tong:** Formal analysis (equal). **Rui Liu:** Formal analysis (equal). **Chengwei Tang:** Conceptualization (equal); Funding acquisition (equal); Supervision (equal); Writing‐review & editing (equal). **Jinhang Gao:** Conceptualization (equal); Funding acquisition (equal); Project administration (equal); Supervision (equal); Writing‐review & editing (equal).

## Supporting information

Supplementary Material

## Data Availability

All the data are present in the manuscript. All the data are available from the corresponding author Jinhang Gao (gjh731@foxmail.com) under reasonable request.
