# Supplementary material for: Celecoxib reduces hepatic vascular resistance in portal hypertension by amelioration of endothelial oxidative stress
Source: J Cell Mol Med. 2021 Oct 5;25(22):10389–402. doi: 10.1111/jcmm.16968 (PMC8581330; doi:10.1111/jcmm.16968)
Supplement: Supplementary file 1 — Supplementary Material [file JCMM-25-10389-s001.docx]

**Supplementary material**

**Supporting Tables**

**Table S1. Primary antibodies used in immunohistochemistry and Western blot**

| **Antibody** | **Application** | **Dilution** | **Source** | **Company** |
| --- | --- | --- | --- | --- |
| GAPDH | WB | 1:2000 | Mouse | Santa Cruz Biotechnology, USA |
| α-SMA | IHC  WB | 1:400  1:1000 | Mouse | Novus Biologicals, USA |
| p-AMPKα  (Thr172) | WB | 1:400 | Rabbit | Cell Signaling Technology, USA |
| AMPKα | WB | 1:1000 | Rabbit | Cell Signaling Technology |
| p-eNOS (Ser1177) | WB | 1:500 | Rabbit | SAB, USA |
| eNOS | WB | 1:1000 | Rabbit | Cell Signaling Technology |
| Nitrotyrosine | WB | 1:400 | Mouse | Santa Cruz Biotechnology |
| NOX1 | WB | 1:400 | Rabbit | Abcam, Cambridge, UK |
| NOX4 | WB | 1:1000 | Rabbit | SAB |
| Dinitropheny | WB | 1:1000 | Mouse | Santa Cruz Biotechnology |
| NRF2 | IHC  WB | 1:200  1:1000 | Rabbit | Proteintech, Wuhan, China |
| KEAP1 | IHC  WB | 1:200  1:1000 | Rabbit | Proteintech |
| HO-1 | IHC  WB | 1:400  1:4000 | Rabbit | Proteintech |
| COX-2 | IHC  WB | 1:400  1:1000 | Rabbit | Abcam |
| COX-1 | IHC  WB | 1:200  1:1000 | Rabbit | HuaAn Biotechnology, Hangzhou, China |
| EP2 | WB | 1:500 | Rabbit | Abcam |
| p-LKB1  (Ser428) | WB | 1:1000 | Rabbit | Cell Signaling Technology |
| LKB1 | WB | 1:1000 | Rabbit | Cell Signaling Technology |

WB, Western blot; IHC, immunohistochemistry; GAPDH, glyceraldehyde-3-phosphate dehydrogenase; α-SMA, alpha-smooth muscle actin; AMPK, AMP-activated protein kinase; eNOS, endothelial nitric oxide synthase; NOX, NADPH oxidase; NRF2, nuclear factor (erythroid-derived 2)-like 2; KEAP1, Kelch-like ECH-associated protein 1; HO-1, heme oxygenase-1; COX, cyclooxygenase; EP2, E-prostanoid receptor 2; LKB1: liver kinase B1.

**Table S2. Primer sequences for qRT-PCR**

| **Gene** | **NCBI Reference Sequence** | **Sequence (5’-3’)** | | **Expected Product**  **Size** |
| --- | --- | --- | --- | --- |
| *Gapdh* | NM_017008 | F | TCGGTGTGAACGGATTTG | 173 bp |
|  |  | R | CTCAGCCTTGACTGTGCC |  |
| *α-SMA* | NM_031004 | F | CCGAGATCTCACCGACTACC | 120 bp |
|  |  | R | TCCAGAGCGACATAGCACAG |  |
| *Vim* | NM_031140 | F | GGAGATGAGGGAGTTGCG | 342 bp |
|  |  | R | GTGAGGTCAGGCTTGGAAA |  |
| *Col1a1* | NM_053304 | F | GATGGCTGCACGAGTCAC | 239 bp |
|  |  | R | GAGTTTGGGTTGTTGGTCTG |  |
| *Col3a1* | NM_032085 | F | GATGGCTGCACTAAACACACT | 241 bp |
|  |  | R | CACTTTCACTGGTTGACGAGA |  |
| *Mmp2* | NM_031054 | F | GACAAGTGGTCCGAGTAAAGTATG | 190 bp |
|  |  | R | GTAAACAAGGCTTCGTGGG |  |
| *Mmp9* | NM_031055 | F | GACGGTCGGTATTGGAAGT | 152 bp |
|  |  | R | TTGCGCCCAGAGAAGAAG |  |
| *Timp1* | NM_053819 | F | CCTCTGGCATCCTCTTGTTG | 294 bp |
|  |  | R | ACTCCTCGCTGCGGTTCT |  |
| *Timp2* | NM_021989 | F | TGAGCGAGAAGGAGGTGG | 348 bp |
|  |  | R | TGTAGCATGGGATCATAGGG |  |
| *Nox1* | NM_053683 | F | ATCACAACCTCACCTTCCATAA | 307 bp |
|  |  | R | CTGCGGATAAACTCCATAGC |  |
| *Nox4* | NM_053524 | F | TGTCCTACTGAAACCAAAGCA | 239 bp |
|  |  | R | AACGGAGTGACCCCAATG |  |
| *Ho-1* | NM_012580 | F | TGACAGAAGAGGCTAAGACCG | 263 bp |
|  |  | R | AATTCCCACTGCCACGGT |  |

qRT-PCR, quantitative real-time PCR; F: Forward; R: Reverse; Gapdh, glyceraldehyde-3-phosphate dehydrogenase; α-SMA, alpha-smooth muscle actin; Vim, vimentin; Col1a1, collagen type I alpha 1; Col3a1, collagen type III alpha 1; Mmp, matrix metalloproteinase; Timp, tissue inhibitor of metalloproteinase; Nox, NADPH oxidase; Ho-1, heme oxygenase-1.

**Table S3. Effects of celecoxib on body weight and organ index**

| **Parameter** | **Control** | **TAA** | **TAA + celecoxib** |
| --- | --- | --- | --- |
| **n** | 12 | 12 | 12 |
| **Initial body weight (g)** | 236.1 ± 7.3 | 233.1 ± 10.4 | 237.8 ± 6.2 |
| **Final body weight (g)** | 571.4 ± 54.1 | 439.2 ± 61.4^*^ | 493.2 ± 62.3^*#^ |
| **Liver**  **(% total weight)** | 2.46 ± 0.23 | 3.21 ± 0.37^*^ | 3.11 ± 0.42^*^ |
| **Spleen**  **(% total weight)** | 0.16 ± 0.02 | 0.28 ± 0.07^*^ | 0.22 ± 0.05^*#^ |

TAA, thioacetamide. Data are presented as mean ± SD. *^*^ p* < 0.05 *vs.* Control group, ^#^ *p* < 0.05 *vs.* TAA group.

**Table S4. Effects of celecoxib on biochemical parameters**

| **Parameter** | **Control** | **TAA** | **TAA + celecoxib** |
| --- | --- | --- | --- |
| **n** | 12 | 12 | 12 |
| **Total bilirubin (μmol/L)** | 1.50 ± 0.24 | 1.53 ± 0.24 | 1.47 ± 0.32 |
| **ALT (IU/L)** | 40.11 ± 10.89 | 46.83 ± 7.94 | 46.86 ± 5.43 |
| **AST (IU/L)** | 132.20 ± 62.33 | 138.14 ± 34.92 | 137.89 ± 60.44 |
| **Albumin (g/L)** | 31.61 ± 1.72 | 33.24 ± 1.39 | 31.77 ± 1.93 |
| **Urea nitrogen (mmol/L)** | 7.36 ± 1.15 | 7.47 ± 0.91 | 7.06 ± 0.72 |
| **Creatinine (μmol/L)** | 35.86 ± 2.34 | 32.67 ± 4.61 | 32.33 ± 3.12 |

TAA, thioacetamide; ALT, alanine aminotransferase; AST, aspartate aminotransferase. Data are presented as mean ± SD.

**Supporting Figures**

**
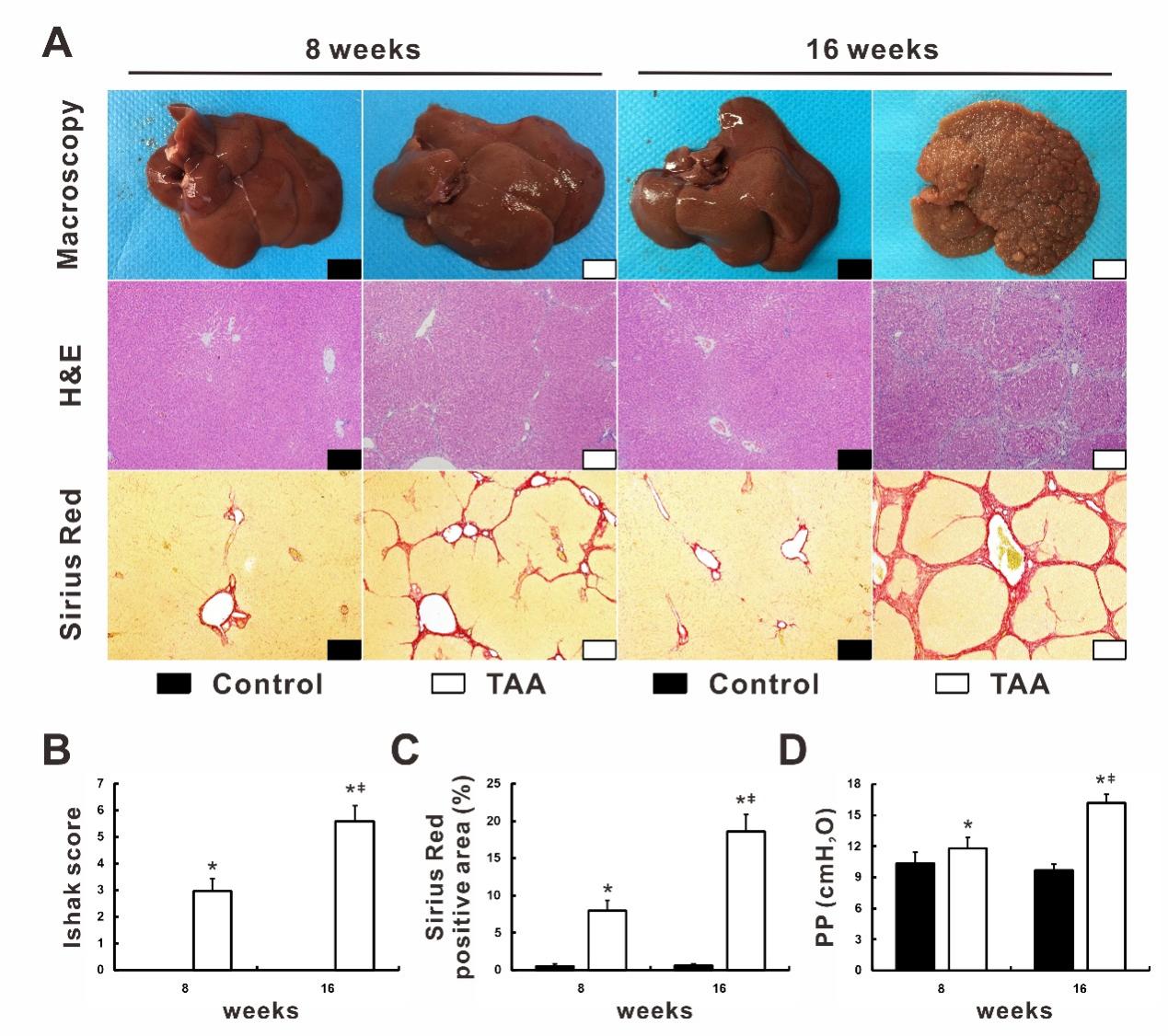
**

**Figure S1. Establishment of liver cirrhosis and portal hypertension.**

*Sprague-Dawley* rats were treated with TAA for 8 or 16 weeks. A-C: Representative liver morphology, histology with H&E and Sirius Red staining (A, ×100 magnification), and quantification of hepatic fibrosis as measured by the Ishak score (B) and fibrotic area (C). D: PP was measured by portal catheterization. Data are presented as mean ± SD. n=12/group, ^*^ *p* < 0.05 *vs.* Control group, ^ǂ^ *p* < 0.05 *vs.* TAA 8 weeks group. H&E: hematoxylin and eosin; TAA, thioacetamide; PP, portal pressure.

**
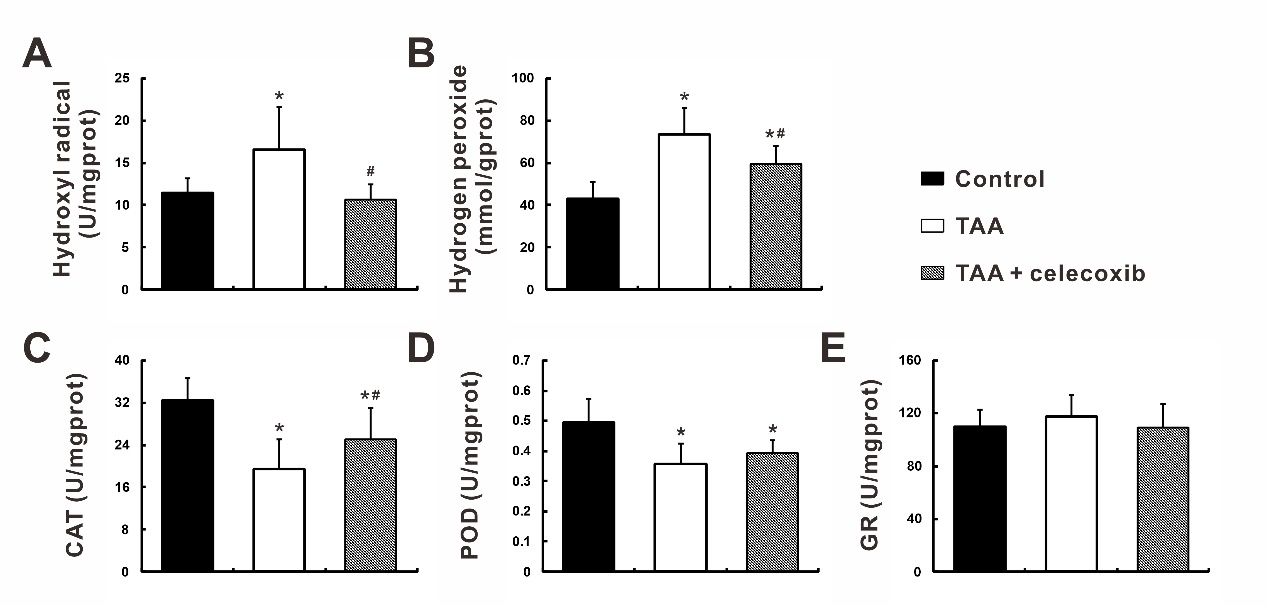
**

**Figure S2. Celecoxib attenuated the imbalance between oxidants and antioxidants in cirrhotic livers.**

A-B: Hepatic oxidative stress was evaluated by hydroxyl radical (A) and hydrogen peroxide (B) levels. C-E: Hepatic anti-oxidative capacity was measured as activities of enzymatic antioxidants CAT (C), POD (D), and GR (E). Data are presented as mean ± SD. n=12/group, *^*^ p* < 0.05 *vs.* Control group, ^#^ *p* < 0.05 *vs.* TAA group. CAT, catalase; GR, glutathione reductases; POD, peroxidase; TAA, thioacetamide.


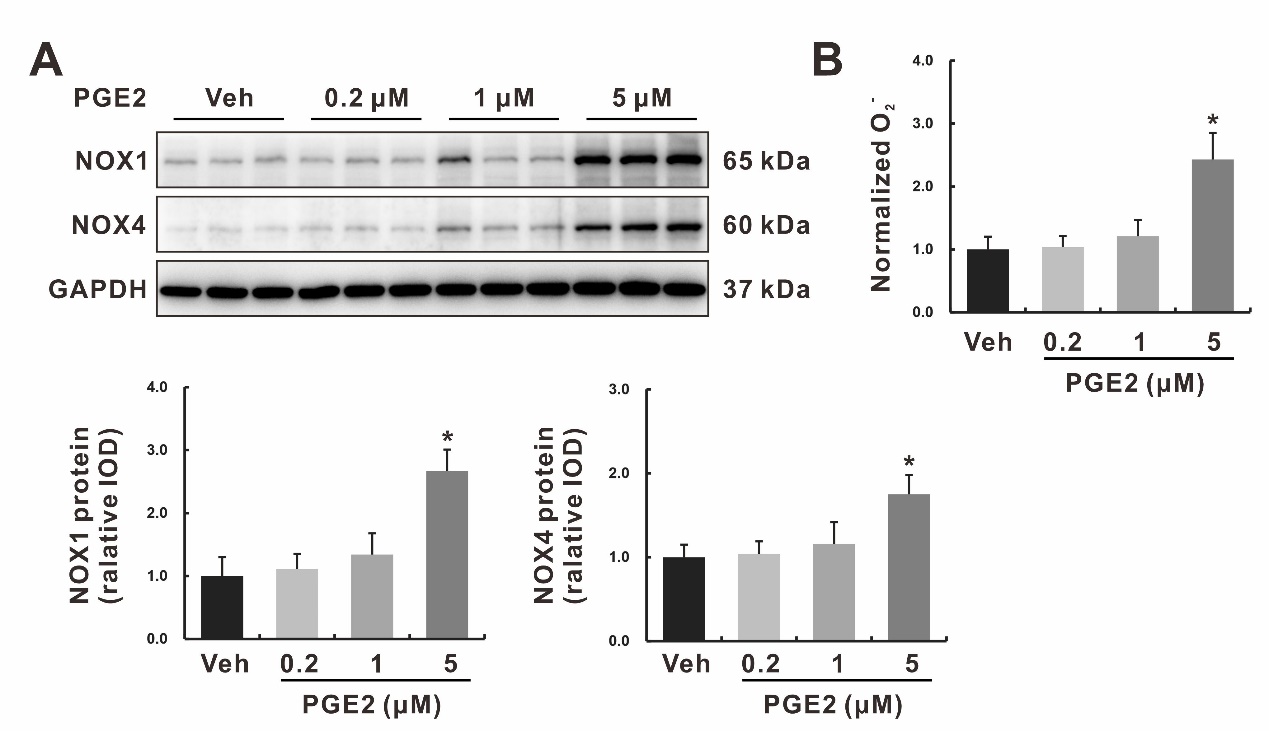


**Figure S3. PGE2 promoted ROS production via up-regulation of NOX1 and NOX4.**

A-B: SK-Hep1 cells were treated with PGE2 for 24 hours. NOX1 and NOX4 protein expression were determined by Western blot (A), and basal ROS production was measured as *in situ* O_2_^-^ levels with DHE fluorescence staining (B). Data are presented as mean ± SD. n=3/group, ^*^ *p* < 0.05 *vs.* vehicle-treated group. GAPDH, glyceraldehyde-3-phosphate dehydrogenase; NOX, NADPH oxidase; PGE2, prostaglandin E2; Veh, vehicle.

**
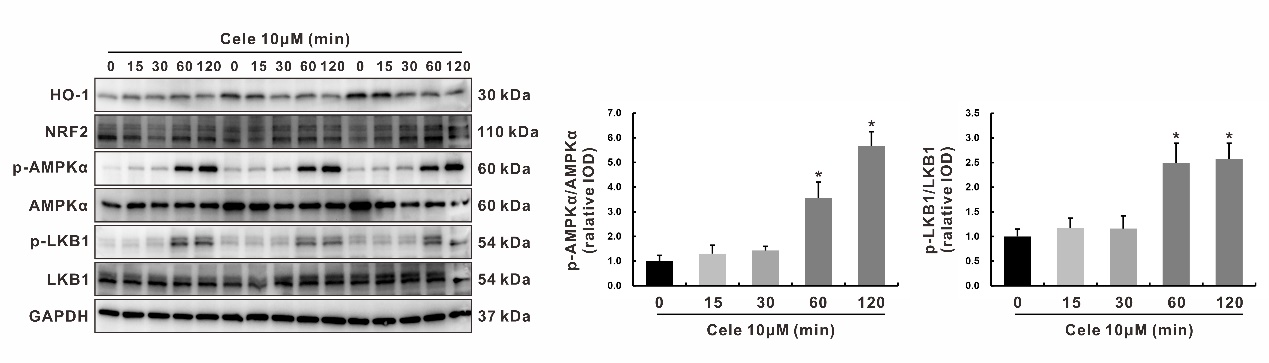
**

**Figure S4.** **Celecoxib activated LKB1-AMPK signaling pathway in SK-Hep1.**

SK-Hep1 cells were treated with celecoxib (10 μM) for up to 120 minutes. Expression of key molecules in the LKB1-AMPK-NRF2-HO-1 signaling pathway were determined by Western blot. Data are presented as mean ± SD. n=3/group, ^*^ *p* < 0.05 *vs.* initial phase. AMPK, AMP-activated protein kinase; Cele, celecoxib; GAPDH, glyceraldehyde-3-phosphate dehydrogenase; HO-1, heme oxygenase-1; LKB1: liver kinase B1; NRF2, nuclear factor (erythroid-derived 2)-like 2.


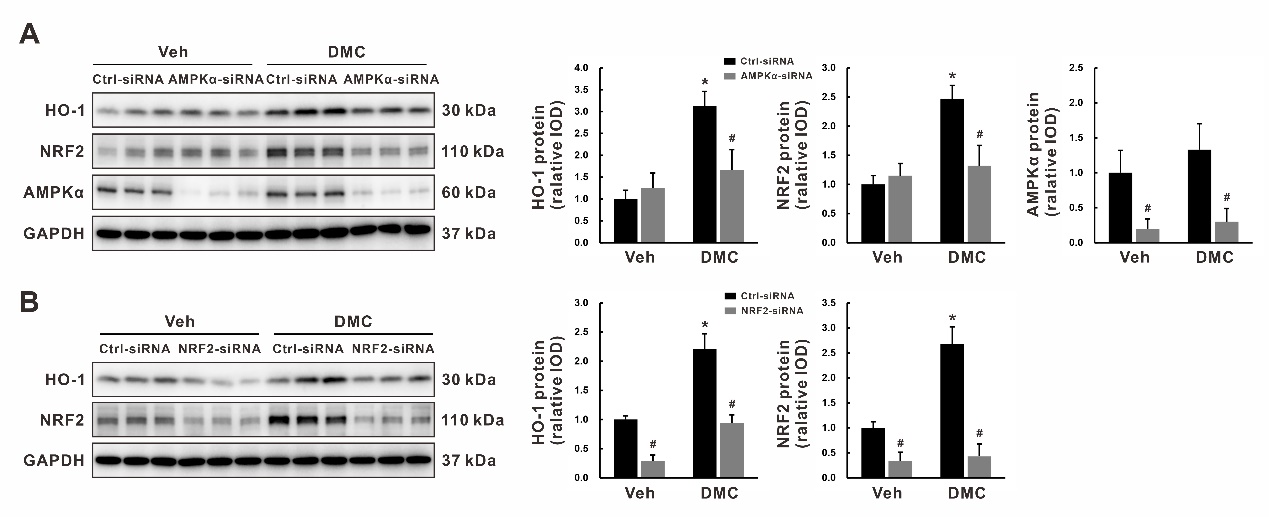


**Figure S5. DMC induced endothelial HO-1 by activating AMPK-NRF2 signaling pathway.**

A-B: SK-Hep1 cells were transfected with AMPKα-siRNA (A) or NRF2-siRNA (B) for 48 hours, followed by induction with DMC (10 μM) for additional 24 hours, expression of key molecules in the AMPK-NRF2-HO-1 signaling pathway were determined by Western blot. Data are presented as mean ± SD. n=3/group, *^*^ p* < 0.05 *vs.* Control-siRNA vehicle-treated group, ^#^ *p* < 0.05 *vs.* Control-siRNA group. AMPK, AMP-activated protein kinase; Ctrl, control; DMC, 2,5-Dimethyl-celecoxib; GAPDH, glyceraldehyde-3-phosphate dehydrogenase; HO-1, heme oxygenase-1; NRF2, nuclear factor (erythroid-derived 2)-like 2; Veh, vehicle.
